# Comparison of the MGIT 960, BACTEC 460 TB and solid media for isolation of *Mycobacterium bovis* in United States veterinary specimens

**DOI:** 10.1186/1746-6148-9-74

**Published:** 2013-04-11

**Authors:** Suelee Robbe-Austerman, Doris M Bravo, Beth Harris

**Affiliations:** 1 United States Department of Agriculture, Mycobacteria and Brucella Section, National Veterinary Services Laboratories, Veterinary Services, Animal Plant Health Inspection Service, Ames, IA, USA

## Abstract

**Background:**

Bacteriologic culture remains one of the most important methods to diagnose bovine tuberculosis despite the lengthy incubation time, significant decontamination and media expense, and high biocontainment requirements. Media selection is an important determination of culture sensitivity, and the planned discontinuation of the BACTEC 460 TB culture system has challenged veterinary diagnostic laboratories to evaluate alternatives. At the National Veterinary Services Laboratories the BACTEC MGIT 960 and 4 solid media formulations were compared with the BACTEC 460 TB system on 6,795 veterinary diagnostic specimens submitted for *Mycobacterium bovis* culture.

**Results:**

*M. bovis* was isolated from 2.6% of the samples and atypical mycobacteria from 4.4% of the samples. The BACTEC 12B media isolated significantly more *M. bovis* (93.1% of positive samples) than MGIT 960 media (81.9%). However, contamination rates were much higher for the MGIT media, 17-24%, compared to 7% for BACTEC, suggesting that contamination was a major cause of MGIT reduced sensitivity. Time to signal positive was 2.37 weeks (95% CI 2.24-2.5) for the MGIT, and 3.2 weeks (95% CI 3.07-3.3) for the BACTEC, both earlier than any solid media. Mycobactosel LJ failed to isolate *M. bovis* from primary culture. An in-house 7H11 media supplemented with calf sera, hemolyzed blood, malachite green and pyruvate recovered more *M. bovis* (80.6%) with the least amount of contamination of any other solid media evaluated.

**Conclusion:**

Decontamination methods may have to be optimized and or MGIT media may have to be altered to reduce contamination in veterinary samples. Despite these issues, the MGIT 960 system is still favored over the use of solid media due to decreased time to recovery and the potential for higher sensitivity.

## Background

*Mycobacterium bovis (M. bovis)*, the cause of bovine tuberculosis (bTB), has a wide host range infecting many species including humans. In 1917 the United States implemented the State-Federal Cooperative Bovine Tuberculosis Program with the goal of eradicating bTB from the US cattle population.

In 1965 the USDA suspended routine antemortem testing requirements for cattle and instead focused primarily on slaughter surveillance and some cattle movement testing [1]. Granulomatous tissue lesions primarily from the head and thoracic cavity are identified at slaughter inspection and submitted to the National Veterinary Services Laboratories (NVSL) for histopathology and, if histology is not able to definitively determine an etiologic cause for the granuloma, the sample is cultured. Approximately 40-45% of slaughter surveillance samples and all antemortem bTB test suspects and reactors that are submitted to NVSL are cultured. Other specimens submitted to the laboratory under the eradication program include cattle necropsied based on exposure to infected animals and wildlife located around previously identified infected cattle herds.

Several studies have evaluated media performance on human specimens for *Mycobacterium tuberculosis* (*M. tuberculosis),* and these studies are nicely summarized in a systematic review [2]. The 2 liquid culture systems most commonly used are the BACTEC MGIT 960 System, which uses the MGIT 960 media (MGIT), and the BACTEC 460 TB System, which uses BACTEC 12B media (BACTEC) (Becton Dickinson and Company, Sparks, MD). While these media systems are comparable, the MGIT typically recovered more mycobacteria other than *M. tuberculosis complex* (MOTT) than the BACTEC, but had higher contamination rates. The recovery of *M. tuberculosis* complex (MTBC) has been mixed, and in general, while not statistically significant, most studies suggest MTBC was recovered more often using BACTEC than the MGIT. Several researchers have suggested increased contamination rates in the MGIT caused the reduced sensitivity, as the laboratories that were able to control contamination rates had improved recovery. Both liquid media systems uniformly outperformed Lowenstein-Jensen (LJ) media in nearly every study [3-5].

The vast majority of veterinary specimens are postmortem, a key difference between routine human and veterinary samples. Little has been published comparing the MGIT, the BACTEC and solid media for veterinary samples. The mycobacterial culture section of NVSL’s Diagnostic Bacteriology Laboratory has used a variety of solid and liquid media formulations over the years for the isolation of *M. bovis* and any other mycobacteria that may interfere with antemortem tests, but a rigorous evaluation of media performance on routine veterinary submissions has not been done. Diagnostic laboratory retrospective studies evaluating media performance while valuable, can have problematic biases such as altering procedures once an isolate is recognized, and preferential sampling of a specific media. Research studies outside of diagnostic laboratory processes also can have significant problems; media may not be quality controlled, technicians or graduate students conducting the testing may be inexperienced or improperly trained, and selected samples may not represent the broader spectrum of samples routinely tested [6].

Our objective was to conduct a prospective study of culture media performance on the routine tissue sample submissions to NVSL. To ensure consistent treatment of samples the study was only initiated after procedures were put in place to require uniform and independent interpretation of media results. Culture media methods evaluated were two liquid culture systems, MGIT and BACTEC, 4 different solid media: 2 in house solid media [modified Middlebrook 7H11 (M7H11P) and Middlebrook 7H10 (7H10P)]; and 2 purchased media, BBL Mycobactosel LJ (M-LJ), and Seven H11 with aspartic acid and pyruvate (7H11AAP).

## Methods

### Clinical specimens

A total of 8,108 specimens were submitted to NVSL for mycobacterial culture during the study period. Out of these, 6,795 specimens were submitted for *M. bovis* culture and included in this analysis. The vast majority of specimens were from cattle, followed by cervids, coyotes/foxes, raccoons/opossums, feral swine, bison, and other ruminants such as goats and sheep throughout the United States (Table 1). Granulomatous lymph nodes from the head and thoracic region were the most common specimen, followed by granulomas from lungs or other organs and grossly normal lymph nodes. If applicable, histology was conducted prior to culture, and the media to be used for inoculation was selected based on histology results and clinical history.

**Table 1:** **Distribution of culture results and species represented in samples submitted for *M. bovis *culture during 2008**

	**Overall culture results**				
**Species**	**No isolation**	***M. bovis***	**MOTT**^**1**^	**Contaminated**	**Grand total**
**Bison**	29	0	6	2	**37**
**Cattle**	4787	71	203	287	**5348**
Cervids*	920	89	52	10	**1071**
**Other Ruminants**^**¥**^	31	0	2		**33**
**Coyotes and Fox**	92	11	8	5	**116**
**Opossums and Raccoons**	113	2	7	7	**129**
**Feral Swine**	31	5	24	1	**61**
**Grand Total**	**6003 (88.4%)**	**178 (2.6%)**	**302 (4.4%)**	**312 (4.6%)**	**6795**

*Species include primarily white-tailed deer, elk, fallow deer, and red deer, both farmed and wildlife.

^**¥**^ Species include sheep, goats and exotic zoo ruminants.

^**1**^ Mycobacteria other than tuberculosis.

### Media used

BACTEC media was prepared by adding double the recommended amount of BACTEC PANTA Plus (400 units Polymyxin B, 40 μg each of amphotericin, trimethoprim and azlocillin and 160 μg of nalidixic acid per BACTEC vial), and 6.0 μg/ml of erythromycin. Inoculated bottles were read twice weekly for the first 3 weeks then once per week for the remaining three weeks. Bottles were not pulled for staining until they reached a Growth Index (GI) reading of 300. All media that had a GI reading of over 25 but less than 300 were stained and read at the end of incubation. Bottles with no GI over 25 were reported as “no isolation made”. If no acid-fast organisms were observed on stain, signal positive bottles were presumptively considered contaminated.

MGIT media was prepared according to manufacturer’s instructions, with the addition of erythromycin 6.0 μg/ml. Inoculated tubes were placed into the MGIT instrument and, if tubes were signal positive, acid fast stains were performed. If no acid-fast organisms were observed, the tubes were incubated in a traditional 37°C incubator until the end of incubation (42 days) and a final acid-fast stain was made. All MGIT tubes that were signal positive, acid fast negative at the end of incubation were presumptively considered contaminated. Tubes that did not signal positive within the 42-day incubation period were recorded as no isolation made.

M7H11P was prepared from Middlebrook 7Hll agar (BD, Sparks, MD) according to manufacturer’s instructions and modified by adding 10% sterile calf serum, 0.5% lysed sheep blood, 0.39% sodium pyruvate, and 0.025% malachite green.

7H10P was prepared from Middlebrook 7H10 agar (BD, Sparks, MD) according to manufacturer’s instructions; except glycerol was not included and 0.41% of sodium pyruvate was added.

M-LJ (BD, Sparks, MD) and 7H11AAP (BD, Sparks, MD) were commercially available media.

Solid media tubes were read weekly for 8 weeks. Colonies morphologically similar to mycobacteria were identified as suspicious and acid fast stained. The media was recorded as contaminated if greater than 50% of the slant was covered by contaminating organisms at the end of 8 weeks, and no acid fast organisms had been identified on the slant.

### Testing procedure

If required by the eradication program, histology was conducted prior to culture. Fresh or borate preserved tissue samples were trimmed of excess fat and connective tissue and soaked for 15 ± 5 min. in 0.065% sodium hypochlorite solution and then macerated using a household blender with a maximum of 50 g tissue and 300 ml of phenol red nutrient broth in a class III biosafety cabinet. Seven ml of macerated tissue and broth were placed in 5 ml of 2% NaOH and decontaminated for 7–10 min and neutralized to effect with 6 N HCL. Specimens were centrifuged at 4800× *g* for 20 min and the supernatant decanted off [6]. Cotton swabs were used to inoculate solid media and 500 μl was pipetted into the liquid media.

All specimens from the same animal were considered high risk if one or more were identified as mycobacteriosis compatible based on histology and consequently were inoculated onto all 6 media. Low risk, non-mycobacteriosis compatible granulomas that arrived to the laboratory preserved in sodium borate were inoculated on to one MGIT and a tube of each of the 4 solid media. Samples submitted fresh or frozen, without preservative were cultured using a liquid only protocol, consisting of a BACTEC and a MGIT vial. A total of 680 samples were inoculated onto all media, 2,488 samples were inoculated using the liquid only protocol, and 3,627 low risk samples were inoculated onto MGIT and solid media only. All media not contaminated or positive were monitored for the recommended incubation period regardless of the results on other media.

Acid-fast organisms were subjected to an FDA approved nucleic acid probe for MTBC (Gen-Probe, San Diego, CA) according to manufacturers’ recommendations. If MTBC positive, spoligotyping and niacin/nitrate biochemical testing were conducted to confirm the isolate was *M. bovis*. Acid-fast positive, nucleic acid probe negative isolates were sequenced using 16S rDNA and blasted against the RIDOM database.

### Data analysis

Results were entered into an Excel spreadsheet from individual case files. To evaluate recovery of *M. bovis*, recovery of atypical mycobacteria and contamination rates for each media, each inoculation protocol (liquid only, low risk- histologically negative, and high risk or histologically positive) was evaluated separately using log linear models to obtain point estimates and 95% confidence intervals with R software. ANOVA was used to compare differences in incubation times between the BACTEC and MGIT, 7H11P and 7H10P.

## Results

A total of 6,795 cases were submitted for *M. bovis* culture during the study period, of which *M. bovis* was recovered from 178 (2.6%). Table 1 lists the animals submitted and summary of culture results. The overall recovery of acid-fast organisms during the study period was 7%. The overall contamination rate of 4.6% was a composite result requiring concurrent contamination on all media.

Individual media contamination rates were significantly higher than the overall rate of 4.6% (Table 2). MGIT media contamination rates varied with sample type with routine slaughter surveillance granulomas having a 1.3 times higher contamination rate compared to fresh/frozen and high risk samples. MIGIT media also had 2.5 times higher contamination rates than the BACTEC media on fresh/frozen and high-risk samples. In contrast to both MGIT and BACTEC, solid media had very high contamination rates (34% and 49%) when inoculated with high-risk samples. Generally, less contamination was reported on the solid media when inoculated with non-mycobacteriosis compatible granulomas submitted in sodium borate.

**Table 2:** Contamination rates for all 3 types of samples processed

	**Contamination rates**		
**Media inoculated**	**Fresh/frozen samples (liquid only)**	**Routine slaughter surveillance histologically non-compatible granulomas**	**Histologically compatible and other high risk samples**
BACTEC 12B	0.07	N/A	0.07
MGIT	0.18	0.24	0.18
M7H11P	N/A	0.14	0.34
7H10P	N/A	0.24	0.49
M-LJ	N/A	0.07	N/A*
7H11AA&P	N/A	0.29	N/A*

Samples submitted fresh or frozen were inoculated only using the 2 liquid media systems. Routine slaughter surveillance samples were inoculated using MGIT and 4 solid media. High risk samples were inoculated on both liquid systems and at least 3 solid media.

* Media results for these samples were not included in the log linear model.

There were 3,168 specimens inoculated concurrently onto both BACTEC and MGIT media including low risk, non-lesioned surveillance wildlife (inoculated using the liquid only protocol) and high risk samples (inoculated onto all media). The results of this comparison are reported in Table 3. The MGIT recovered more acid-fast bacteria than the BACTEC (269 vs. 252). Of the 178 specimens where *M. bovis* was recovered, 172 were included in this sample set, of which liquid media failed to detect 3. The BACTEC significantly outperformed the MGIT for *M. bovis* recovery (161 vs. 144).

**Table 3:** Cross tabulation comparison of all specimens inoculated simultaneously on to both BACTEC and MIGIT media

		**All cases**^**a**^				***M. bovis***^**b**^				**MOTT**^**c**^			
		**MGIT**				**MGIT**				**MGIT**			
		**AF+**	**Neg**	**Cont.**	**Total**	**AF+**	**Neg**	**Cont.**	**Total**	**AF+**	**Neg**	**Cont.**	**Total**
**BACTEC**	**AF+**	179	33	40	**252**	136	5	20	**161**	43	28	20	**91**
	**Neg**	79	2217	394	**2690**	6	1	2	**9**	73	9	3	**85**
	**Cont.**	11	83	132	**226**	2	0	0	**2**	20	9	0	**13**
		**269**	**2333**	**566**	**3168**	**144**	**6**	**22**	**172**	**125**	**37**	**27**	**189**

^a^ Includes all cases tested using both MGIT 960 media and BACTEC 12B media.

^b^ Includes only cases where *M. bovis* was recovered, and both MGIT 960 media and BACTEC 12B media were used. There were 3 samples were *M. bovis* was recovered only on solid media.

^c^ Includes only cases where atypical mycobacteria were recovered, and both MGIT and BACTEC media was used. There were 21 samples where atypical mycobacteria were recovered on solid media only.

A total of 160 *M. bovis* positive samples were inoculated on to both liquid and solid media and are compared in Table 4. Based on the log linear models, all results were statistically significantly different from each other except for MGIT and M7H11P. M7H11P was the best performing solid media. Mycobactosel LJ failed to grow *M. bovis* on primary inoculation. That media did however recover 3 atypical mycobacteria from *M. bovis* positive specimens. 7H11AAP was very clearly underperforming, not only having high contamination rates, but also failing to grow *M. bovis* when there was luxuriant growth on other Middlebrook media. Because of the expense, this media was discontinued early and was not included in the log linear model to estimate sensitivity.

**Table 4:** **Comparison of the media on the 160** ***M. bovis *positive tissues that were inoculated onto all media**

	***M. bovis *+ No. (%)**	**Negative No. (%)**	**Contaminated No. (%)**	**Not tested**
BACTEC	150 (93.8)	8 (5.0)	2 (1.3)	
MGIT	131 (81.9)	6 (3.8)	23 (14.4)	
M7H11P	129 (80.6)	11(6.9)	20 (12.5)	
7H10P	104 (65.0)	16 (10.0)	40 (25.0)	
M-LJ	0	152 (95.0)	8 (5.0)	
7H11AAP	24 (27.9)	45 (52.3)	17 (19.8)	74

The difference in sensitivity recovering *M. bovis* between the BACTEC and MGIT, and even some of the solid media, seemed to be directly related to the contamination rates of the media. In an attempt to statistically evaluate that observation, pairwise comparisons were made within the log linear model for each media type given that *M. bovis* was recovered from at least one media. It was found that the MGIT contamination rate was statistically significantly higher than the BACTEC (Difference of 0.13; 95% CI: 0.06, 0.21) and 7H11P was statistically significantly higher than BACTEC (Difference of 0.12; 95% CI: 0.05, 0.19). All other comparisons did not reveal statistically significant differences.

**Figure 1:**
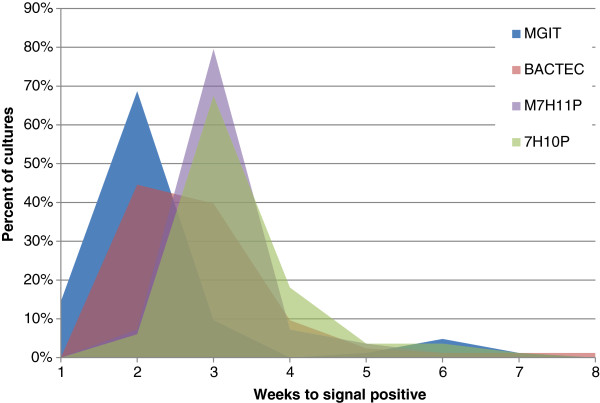
**Comparison of BACTEC and MGIT, M7H11P and 7H10P times to detection for 83 isolates where recovery of *M. bovis *was successful on all media.**

The average time to detection was 2.37 weeks (95% CI 2.18-2.55) for the MGIT, 3.2 weeks (95% CI 3.02-3.37) for the BACTEC, 3.38 weeks (95% CI 3.22-3.53) for M7H11P, 3.5 weeks (95% CI 3.35-3.70) for 7H10P, and 4.0 weeks (95% CI 3.5-4.5) for 7H11AA&P. To directly compare times to detection, 83 samples where MGIT, BACTEC, M7H11P, and 7H10P concurrently recovered *M. bovis* were used. MGIT was the only media to recover *M. bovis* within one week (14.5% of the time) and by week 2 had recovered 83.1% compared to the other media at week 2: BACTEC at 44.6%, M7H11P at 7.2% and 7H10P at 6% (Figure 1). M7H11P and 7H10P were the only 2 media not significantly different in time to detection.

While the focus of this paper was *M. bovis* recovery, differences in recovery of atypical bacteria were also observed. Table 2 also included the results of the BACTEC and MGIT for recovery of atypical mycobacteria. Of the 189 samples, the MGIT recovered 125 atypical mycobacteria (66% sensitivity) vs. 91 from the BACTEC(48% sensitivity). There was low agreement as only 43 were recovered simultaneously from both media. Liquid media systems identified 2–10 times more atypical mycobacteria than solid media.

## Discussion

The discontinuation of the BACTEC has challenged veterinary mycobacteriology laboratories to investigate different liquid media formulations. For many years, the BACTEC has been the mainstay of many veterinary laboratories including NVSL. In this study, using a decontamination protocol that clearly was effective for BACTEC (93.8% recovery rate of positive specimens, 7% contamination rate) does not appear to be optimized for the MGIT system (81.9% recovery rate, 18% contamination rate). Although this is simply an observational study, the data published here suggest that contamination was a major reason for reduced sensitivity of the MGIT media.

Higher contamination rates in the MGIT compared to the BACTEC are a consistent finding in the literature [7-9]. The primary difference between MGIT and BACTEC is that MGIT media contains dextrose replacing the radioactive palmitic acid found in BACTEC. The addition of the dextrose reduces the selectivity of the media [7]. Despite the richness of the MGIT media, it may be possible to reduce contamination by altering some procedures. Examples include increasing the harshness of the decontamination method or increasing the amount of antibiotics. In this study, the laboratory was using a 7–10 minute decontamination protocol in a 0.8% final concentration of NaOH, which is likely not sufficient for the less selective MGIT media. Clinical Laboratory Standards Institute (CLSI) in their approved guideline recommends up to 2% NaOH, so it is likely the laboratory could increase the concentration of NaOH without negatively impacting its ability to grow mycobacteria [10]. Furthermore, PANTA antibiotic supplement was double the recommended level in the BACTEC 12B but was left at the manufacturer’s recommended concentration in the MGIT media. Since the MGIT PANTA can be purchased separately from the growth supplement, it is reasonable to evaluate increasing antibiotic levels.

The overall MGIT contamination rate of 17% or higher was different than in a previous publication by NVSL, where MGIT contamination rates were reported to be 6.9% [6]. There were key differences in the 2 studies. First Hines et al. used samples from cattle with very high levels of *M. bovis*, collected at a single location. Secondly MGIT media were reported as contaminated only if fungal or bacterial contamination was noted on the acid fast slide. In this study, we considered all signal-positive, acid-fast negative MGIT tubes as contaminated, which in our experience is more accurate as the majority of these tubes readily grow contaminates if subcultured on blood agar plates, even if contaminants are not visible in the acid-fast slide.

The only egg based media used in this study, M-LJ failed to grow *M. bovis* on primary culture. Of course, the requirement for pyruvate supplementation of LJ media for *M. bovis* is well documented in the literature, so it is not particularly surprising that *M. bovis* would fail to grow on a media not containing pyruvate [11].

A simple 7H10 media with pyruvate substituted for glycerol recovered *M. bovis* only 65% of the time, and had significant levels (25.5%) of contamination. The more elaborate M7H11P media outperformed this and all the other solid media with an 80% recovery rate. The addition of hemolyzed red cells, serum and malachite green was likely the cause. This formula was first published in 1977 when hemolyzed sera was found to be a significant growth enhancement to 7H11 [12]. The purchased 7H11AAP media was disappointing, recovering *M. bovis* only 28% of the time from positive tissues.

The MGIT media recovered more MOTT than BACTEC 12B, however, based on the lack of supporting histological evidence in the majority of MOTT culture positive samples, MOTTs were likely most often an incidental finding. Lack of agreement between the liquid media systems when MOTT were recovered also suggests low or sporadic levels of incidental mycobacteria. Nonetheless, broad varieties of mycobacteria were recovered, with a very similar population structure reported elsewhere (Thacker *et. al*.: *Isolation of Mycobacteria from clinical samples collected in the United States from 2004 to 2011*. Forthcoming in BMC Vet Res).

## Conclusions

Veterinary mycobacterial laboratories that previously used BACTEC media will likely have to alter their decontamination procedures and add antibiotics or other supplements to MGIT media in order to reduce contamination and improve the recovery of *M. bovis* from routine veterinary samples. Despite these issues, the MGIT system is still favored over the use of solid media due to decreased time to recovery and higher sensitivity.

## Competing interests

The authors declare that they have no competing interests.

## Authors’ contributions

SRA, DMB, BH conceived the study. DMB reviewed the cases, SRA compiled the results, and BH managed the laboratory. SRA wrote the manuscript and conducted the analysis. All authors participated in the interpretation of the results. All authors read and approved the final manuscript.
